# DREADD agonist compound 21 causes acute diuresis in wild-type mice

**DOI:** 10.3389/fphar.2024.1471059

**Published:** 2024-10-24

**Authors:** Bryce MacIver, Ali Wu, Warren G. Hill, Weiqun Yu

**Affiliations:** Department of Medicine, Beth Israel Deaconess Medical Center and Harvard Medical School, Boston, MA, United States

**Keywords:** compound 21, diuresis, glomerular filtration rate, antagonism, smooth muscle contractility

## Abstract

The targeted activation or inhibition of specific cell populations using chemogenetics allows the precise dissection of cellular signaling and function. Designer receptors exclusively activated by designer drugs (DREADDs) is a chemogenetic platform initially developed by mutating human muscarinic receptors to be unresponsive to endogenous acetylcholine but exclusively activated by an “inert” designer drug. Compound 21 (C21) is a new and potent DREADD agonist; however, radioligand assays from a recent report indicated its ability to bind to endogenous G protein-coupled receptors (GPCRs), including muscarinic M1–3 receptors. Whether this binding causes off-target effects is unclear. Renal innervation is important for the regulation of renal function, and the advent of chemogenetic tools provides significant opportunities for the mechanistic understanding of renal innervation and function. GPCRs such as adrenergic and muscarinic receptors play a role in renal function; thus, a careful pharmacological characterization of C21 in renal function is a prerequisite for this approach. Unexpectedly, an infusion of 1.0 mg/kg C21 in anesthetized mice caused an ∼4-fold increase in urine output and correspondingly increased the glomerular filtration rate (GFR), suggesting a C21-mediated acute diuretic effect. This acute diuresis effect was further confirmed in awake mice using voiding spot assays. The exact molecular mechanism for C21-mediated diuresis is unclear; however, we demonstrated by *in vitro* myography that C21 can effectively inhibit bladder smooth muscle contraction by antagonizing M3 receptors at the micromolar level, causing increased voiding size *in vivo*. In summary, C21 functions as a GPCR antagonist and has significant dose-dependent off-target effects in the renal system.

## Introduction

Optogenetics and chemogenetics are powerful approaches for the targeted activation or inhibition of specific cell populations, allowing the decoding of precise cellular signaling pathways and the functional consequences of activation or inhibition (Sternson and Roth, 2014). Designer receptors exclusively activated by designer drugs (DREADDs) is a chemogenetic platform that was initially developed by mutating human muscarinic receptors (hM3Dq and hM4Di) to be unresponsive to endogenous acetylcholine but exclusively activated by an “inert” designer drug clozapine N-oxide (CNO). Therefore, the strict selectivity of the designer drug for the designer receptor is of critical importance for chemogenetic experiments. Although CNO has been widely used in chemogenetic studies, it has very low permeability across the blood–brain barrier, which limits its accessibility to neuronal tissue and requires a high dosage for this purpose. In addition, recent studies found that CNO can be converted into clozapine *in vivo*, which exhibits high affinity to DREADD receptors and endogenous muscarinic receptors, thus raising the question of whether CNO mediates chemogenetic effects by clozapine (Gomez et al., 2017). A recent report has also shown that CNO can bind to many endogenous G protein-coupled receptors including muscarinic M1–3 receptors, thus raising the question of potential off-target effects (Jendryka et al., 2019). Compound 21 (C21) is a new and potent DREADD agonist derived from CNO; it has a higher affinity to hM3Dq (EC_50_ = 1.7 nM) than CNO (EC_50_ = 6.0 nM) and is permeable across the blood–brain barrier (Thompson et al., 2018). However, radioligand assays also indicated its ability to bind to many endogenous G protein-coupled receptors including muscarinic M1–3 receptors, thus presenting the potential for off-target effects as well (Jendryka et al., 2019).

Renal innervation is important for renal functioning and regulates renal blood flow, glomerular filtration, and ion and water reabsorption. Renal sympathetic innervation is well-established and involves activating adrenergic receptors in the kidney. This mechanism leads to the upregulation of the renin–angiotensin–aldosterone system (RAAS), which is important for the kidney’s ability to regulate ion/water homeostasis and systemic blood pressure (Osborn et al., 2021). Although the cholinergic agonist acetylcholine has long been known to regulate kidney function, potentially by activating muscarinic receptor M3 (Eltze et al., 1993; Wierema et al., 1997; Blankesteijn et al., 1993; Yun et al., 1993), renal parasympathetic innervation is still under debate. A recent study has provided strong evidence that cholinergic nerves supply the renal vasculature and renal pelvis, and a vagal brain–kidney axis is involved in renal parasympathetic innervation (Cheng et al., 2022). The mechanistic understanding of renal innervation and its regulation is still very limited, and the advent of chemogenetic and optogenetic approaches poses great potential to push forward this boundary. It is therefore necessary to rigorously characterize the potential use of these novel DREADD agonists in the renal system.

## Methods

### Materials

Unless otherwise specified, all chemicals were obtained from Sigma (St. Louis, MO) and were of reagent grade or better. α, β-meATP (Tocris, Catalog#: 3209), CNO (Tocris, Catalog#: 6329), and C21 (Tocris, Catalog#: 6422) were purchased from R&D Systems (Minneapolis, MN, United States).

### Animals

Both wild-type male and female C57BL/6J mice (Jackson Laboratory, Bar Harbor, ME) aged 12–20 weeks old were used in this study. All animal studies were performed in adherence with the US National Institutes of Health guidelines for animal care and use and with the approval of the Beth Israel Deaconess Medical Center Institutional Animal Care and Use Committee (Protocol #:066-2022). The mice were housed in standard polycarbonate cages and maintained on a 12:12-h light–dark cycle at 25°C with free access to food and water.

### Mouse femoral artery and vein catheterization

Under 2% isoflurane anesthesia, an ∼1.5-cm incision was made on the inner side skin of a male mouse’s left leg to expose the femoral artery and vein. Using a dissecting microscope (Olympus SZ60), the femoral artery and vein were then carefully isolated. A loose silk knot was made on the proximal end of the femoral artery to temporarily block the circulation, and a fine snip was then made on the distal side of the femoral artery for catheterization. A PE-10 tube with a beveled tip was then inserted into the femoral artery and secured with a silk suture (size 4-0 suture). For the catheterization of the femoral vein, the distal end was tied with a silk knot to block the circulation, and a similar operation was performed.

### Mouse bladder catheterization

Bladder catheterization was performed right after the femoral artery and vein catheterization. A 1-cm midline abdominal incision was made. A flame-flanged PE-50 tubing sheathing a 25 × 1.5-inch needle was implanted through the dome of the bladder and then sutured in place (4-0 silk suture). The incision site was sutured around the tubing using sterile 5-0 silk. A 0.5-mL Eppendorf tube was used to collect the urine from the inserted PE-50 tubing. The volume of urine in the tube was measured approximately every 15 min to obtain at the kidney flow rate.

After the surgery and removal of isoflurane, mouse anesthesia was maintained by infusion with thiobutabarbital via the femoral vein at a dose of 1.4 mg/kg. Thiobutabarbital was prepared at 36 mg/mL in water to be near isotonic.

### Blood pressure and heart rate monitoring and solution infusion

The catheter inserted into the femoral artery was connected to a modified pressure transducer (Catalog #: BLPR2) coupled to data-acquisition devices (WPI Transbridge and ADInstruments PowerLab 4/35), and the blood pressure and heart rate were recorded using a computerized recording system (LabChart software).

The catheter inserted into the femoral vein was connected to a programmable syringe pump (BS-8010, Braintree Scientific, MA) for infusion. To determine the glomerular filtration rate (GFR), a 2.5 mg/mL FITC-sinistrin (MediBeacon, Creve Coeur, MO) solution containing 16.8%v/v phosphate-buffered saline (2%w/v glucose, 2 mg/mL thiobutabarbital, 10 mg/mL BSA, and 10 mg/mL para-amino hippurate) was continuously infused by the femoral vein. The solution was filtered through a 0.22-µm syringe filter before administering to the animal.

C21 dihydrochloride was dissolved in dextrose 5% in water (D5W) to reduce adding further sodium to the system and infused by the femoral vein.

### Renal function analysis

At the end of the experiment, the femoral artery catheter was disconnected from the pressure transducer to bleed the mouse blood into a 2-mL blood collection tube. Plasma and urine sodium concentrations were then measured by flame photometry (BWB, Newbury, United Kingdom) using 100 ppm Li as an internal standard.

The GFR was calculated by measuring the FITC fluorescence of urine and plasma samples using a Molecular Devices (San Jose, CA) SpectraMax M5 instrument. The fractional excretion of sodium (FeNa) was calculated using the GFR. FeNa = Na(urine) × flow rate/Na(plasma) × GFR.

### Myography and electrical field stimulation

Bladders from male mice were pinned on a small SYLGARD block, and muscle was dissected free of the mucosal tissue. Bladder smooth muscle (BSM) strips were then cut longitudinally (2–3-mm wide and 5–7-mm long). The muscle strips were mounted in an SI-MB4 tissue bath system (World Precision Instruments, Sarasota, FL, United States). Force sensors were connected to a TBM 4M transbridge (World Precision Instruments), and the signal was amplified by PowerLab (ADInstruments, Colorado Springs, CO, United States) and monitored through Chart software (ADInstruments). The BSM strips were gently pre-stretched to optimize the contraction force and then pre-equilibrated for at least 1 h. All experiments were conducted at 37°C in physiological saline solution (in mM: 120 NaCl, 5.9 KCl, 1.2 MgCl_2_, 15.5 NaHCO_3_, 1.2 NaH_2_PO_4_, 11.5 glucose, and 2.5 mM CaCl_2_) with continuous bubbling of 95% O_2_/5% CO_2_. The contraction force was sampled at 2,000/s using Chart software. The BSM tissue was subjected to electrical field stimulation (EFS) to mimic *in vivo* neurotransmitter release and examine the general BSM functionality. The BSM strips were then treated with either C21 or CNO to examine whether these compounds have any effect on BSM contractility. Then, 10 μM atropine and 10 µM α, β-meATP were used to examine BSM muscarinic and purinergic contractility. BSM-strip EFS was carried out using a Grass S48 field stimulator (Grass Technologies, RI, United States) using standard protocols previously described: voltage, 50 V; duration, 0.05 ms; trains of stimuli, 3 s; and frequencies 1, 2, 5, 10, 20, and 50 Hz (Chen et al., 2020; Hao et al., 2019). There was a 3-min interval between each stimulus (Yu et al., 2013).

### Voiding spot assay

Dominant male mice typically communicate hierarchy and territoriality through urinary marking, which confounds the interpretation of the voiding spot assy (VSA) data. Therefore, VSAs were performed only on female mice, as described previously (Yu et al., 2014). Prior to the assay, the mice were anesthetized with isoflurane (5% induction and 2% maintenance) and then injected subcutaneously with either D5W (5% dextrose in water) or one of three concentrations of C21 (0.3, 1.0, or 3.0 mg/kg). Anesthesia was used to prevent the mice from urinating during handling and injection. Following injection, the mice were immediately placed individually in empty mouse cages with a pre-cut filter paper (Blicks Cosmos blotting paper #10422-1005) on the bottom. They were provided food in the usual wire racks but no water. Assays were run for either 90 min or 4 h, after which the mice were returned to their home cages, and the filter paper was allowed to dry before being photographed under UV light (365 nm) in a Chromato-Vue C-75 imaging box with onboard Canon camera (EOS Rebel T3–12 megapixels). During image analysis, any overlapping spots were outlined using the drawing tool in the Fiji version of ImageJ (Schindelin et al., 2012), copied, and moved to an empty area of the filter. A machine learning algorithm developed in-house (ML-UrineQuant) and trained on 60 void spot filter images allowed the unbiased quantitation of the total urine volume, the number of primary void spots (PVS: urine spot volume >20 μL), and the mean PVS volume.

### Statistical analyses

The minimum sample number in each experimental group was 5 based on previous experiments with the proposed experimental design. The voiding spot assay needs ∼10 animals in each group due to the relatively large standard deviations observed with the PVS number and PVS volume.

All data are expressed as the mean ± SD or presented as boxes and whiskers (extending from minimum to maximum values). Data were analyzed by Student's *t*-test between two groups. If possible, a paired *t*-test was used. Bonferroni’s multiple comparison *post hoc* tests were used where necessary, and *p* < 0.05 was considered significant. For myography studies, a minimum of three animals were used, and each bladder usually yields 3–4 valid muscle strips, and Student’s *t*-test was used for this experiment.

## Results

### C21 causes acute diuresis in catheterized and anesthetized male mice

**FIGURE 1:**
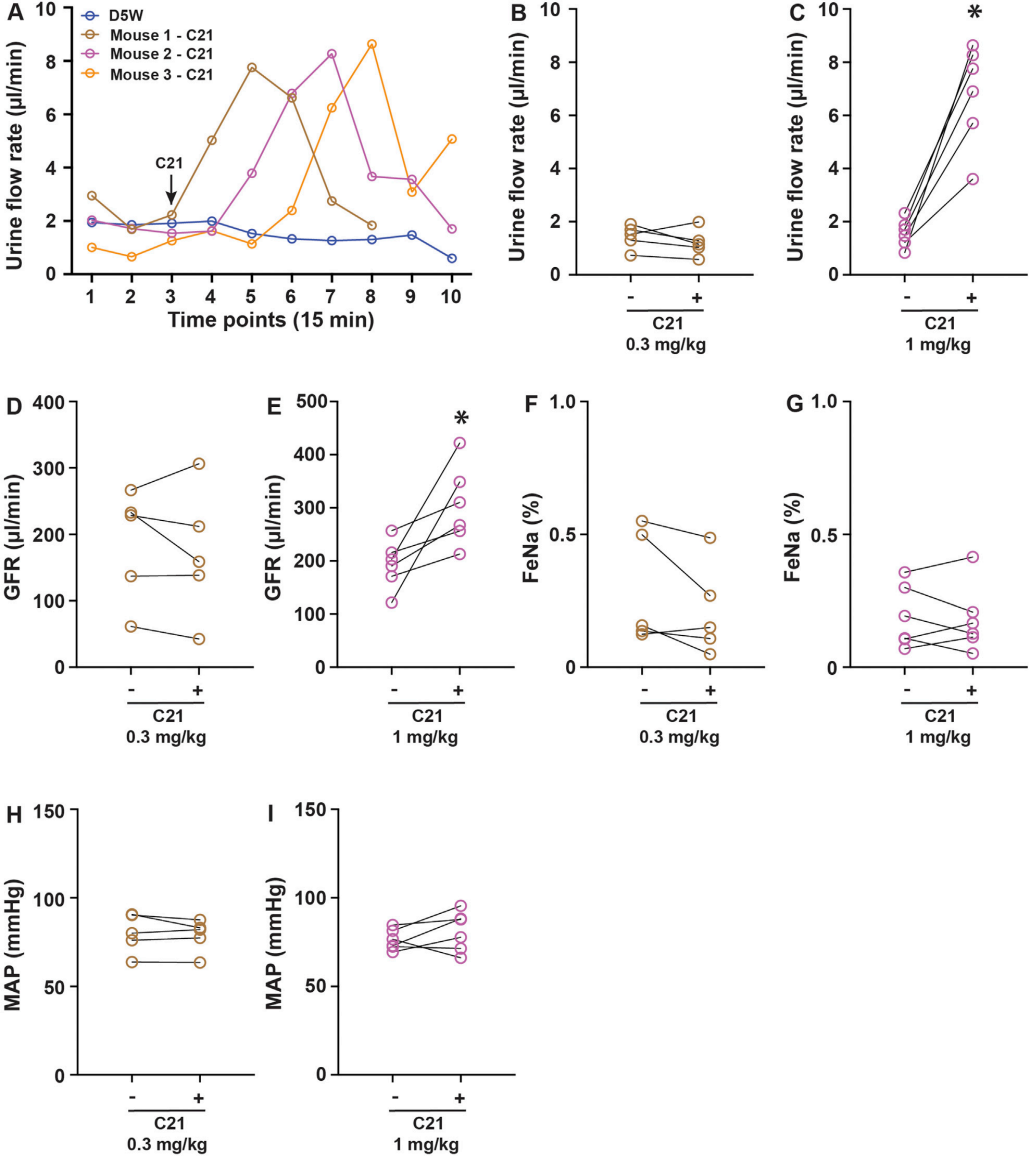
C21 causes acute diuresis in anesthetized mice. **(A)** Shows the representative traces of the urine flow rate in response to control solution D5W and 1 mg/kg C21 infusion, indicating an acute increase and return of urine output in response to 1 mg/kg C21. The quantitated data are shown in **(B,C)**. The administration of C21 significantly increased the GFR **(D,E)** but not FeNa **(F,G)** and MAP **(H,I)**. Data before and after intervention are shown as individual symbols and line plots from each animal. Data were analyzed by paired Student’s *t*-tests. *p* < 0.05 is considered to be significant. The number of mice > or = 5 for each condition.

To evaluate the potential biological effect of C21 in regulating renal function, the anesthetized mice were cannulated in the femoral vein for infusion and the artery for blood pressure monitoring. A solution incorporating FITC-sinistrin for GFR measurement was continuously infused at 120 μL/h/10 g body weight to establish a steady urine output. Urine collections were made every 15 min via a bladder catheter. Typically, 2–3 stable collections were made before an intervention. In chemogenetic studies, C21 was typically administered at 0.3–3 mg/kg, which is considered to be effective in activating engineered hM3Dq and hM4Di receptors without significant off-target effects (Thompson et al., 2018). We administered C21 at 0.3 mg/kg and 1.0 mg/kg (diluted in 100 µL isosmotic D5W solution). It was apparent that 1.0 mg/kg of C21 significantly increased the urine output, which lasted approximately 1 h, with the peak flow rate increased by ∼4-fold compared to the control groups (D5W) or before C21 treatment (Figures 1A–C). Interestingly, this acute diuresis effect was not observed in 0.3 mg/kg C21-infused and D5W-infused animals. The GFR upon 1.0 mg/kg of C21 administration was also significantly increased but not in response to 0.3 mg/kg of C21 dosage (Figures 1D, E). Interestingly, the FeNa and mean atrial pressure (MAP) did not show significant differences in both dosages tested (Figures 1F–I). These data suggest that C21 infusion at 1.0 mg/kg causes acute diuresis by an unknown mechanism, which is an unexpected and unreported C21-mediated effect.

### C21 inhibits male mice’s bladder smooth muscle muscarinic contractility *in vitro*

**FIGURE 2:**
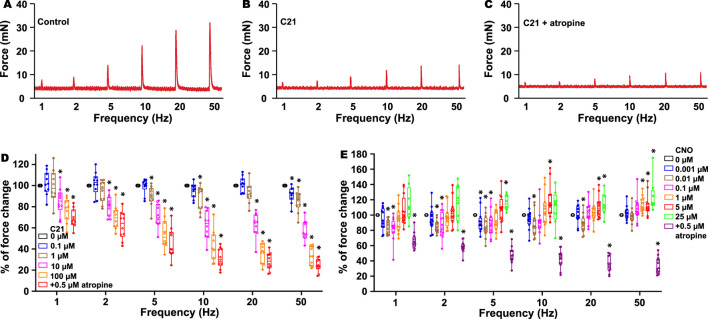
C21 dose-dependently inhibits BSM contraction force. **(A)** is a representative trace of mouse BSM contraction in response to EFS, which is inhibited by C21 **(B)**, and the further addition of atropine only slightly inhibits BSM contraction force **(C)**. **(D)** Summarized data showing that C21 dose-dependently inhibits BSM muscarinic contractile force (n = 10 BSM strips). **(E)** Summarized data showing the biphasic effects of CNO on BSM contractile force in response to EFS (n = 12 BSM strips). Data are shown as box and whiskers, the center line is the median of the dataset, the box represents 75% of the data, and the bars indicate whiskers from minimum to maximum. Data were analyzed by one-way ANOVA with Bonferroni’s *post hoc* tests. *p* < 0.05 is considered to be significant.

As previous radioligand assays have indicated that C21 binds to muscarinic receptor M3 (Jendryka et al., 2019) and it is also known that the activation of the M3 receptor in the kidney regulates diuresis (Eltze et al., 1993; Wierema et al., 1997; Blankesteijn et al., 1993; Yun et al., 1993), we hypothesized that the M3 receptor could be one of the C21 targets to cause diuresis. In the urinary system, M3 receptors also play a critical role in mediating BSM contraction, and the activation of the M3 receptor mediates bladder muscarinic contractility (Yu et al., 2013). To investigate this possibility, we performed a myography study using BSM strips. Interestingly, the addition of C21 did not induce any BSM contraction, indicating that C21 is not an agonist for the endogenous mouse M3 receptors. However, starting from 0.1 µM, C21 dose-dependently inhibited EFS-induced BSM contraction and almost completely inhibited BSM muscarinic contractility at 100 µM (Figures 2A–D). These data suggest that C21 could function as an effective mouse M3 receptor antagonist.

Because C21 is a derivative of CNO and radioligand assays also indicated that CNO binds to muscarinic M1–3 receptors (Jendryka et al., 2019), we further tested whether CNO has an antagonistic effect on M3 receptors. As expected, the addition of CNO at a dose ranging from 1 nM to 25 µM did not induce any contractile activity, indicating that CNO is not an agonist for the endogenous M3 receptors. However, CNO at a concentration of approximately 10 nM significantly inhibited EFS-induced BSM contraction force, and this effect is even more significant at a lower EFS frequency (Figure 2E). In contrast, CNO at concentrations 5–25 µM significantly potentiated high-frequency EFS-induced BSM contraction force (Figure 2E). These complex biphasic effects indicate that CNO might bind to different off-target receptors with different affinities to regulate BSM contractile function.

### C21 causes diuresis and altered void function in unrestrained awake female mice

**FIGURE 3:**
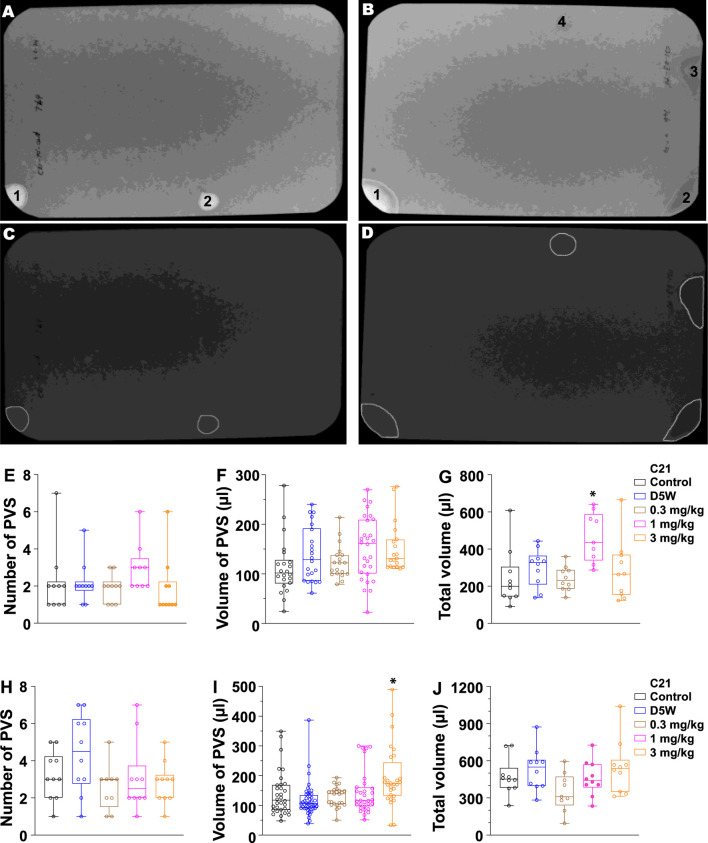
Quantitated void spot patterns on the filter paper demonstrate an acute diuretic effect of 1.0 mg/kg C21 at 90 min and an increase in volume/void at 3.0 mg/kg in 4-h assays. **(A,B)** Void spot patterns after 90 min in **(A)** a control mouse and **(B)** a C21-injected mouse (1.0 mg/kg). **(C,D)** Same filters with spots identified using ML-UrinQuant software. **(E)** 90-min primary void spots of 10 mice subjected to no injection (control) or s.c. injection with D5W, 0.3 mg/kg C21, 1.0 mg/kg C21, and 3.0 mg/kg C21. **(F)** Volume of PVS in 90 min. **(G)** Total voided volume in 90 min. **(H–J)** Same parameters after 4 h. Box and whiskers show the mean ± interquartile range (box) and max–min (whiskers). Data were analyzed by ANOVA with Tukey’s multiple comparison test; *p < 0.05; n = 10 mice/condition.

To further determine whether C21 affects renal and urologic function in free-moving awake mice, we performed voiding spot assays. In this experiment, we administrated C21 by i.p. with three dosages: 0.3, 1, and 3 mg/kg. Because C21-mediated diuresis in anesthetized mice only lasted for ∼1 h, a 90-min VSA method was used. As shown in Figure 3G, a significantly increased total urine volume output was observed at the dosage of 1 mg/kg; however, the total urine volume for 3 mg/kg was not significantly increased. We hypothesized that, given the bladder muscle inhibition caused by C21, the primary voids of the mice would be larger at a dosage of 3 mg/kg of C21, and this could reduce the voiding frequency and thereby reduce the total urine volume at 3 mg/kg of C21 in such a short time. Accordingly, we performed a 4-h VSA. As expected, the volume for primary voids at 3 mg/kg dosage was significantly larger than that in other groups (Figure 3I), indicating that C21 at this dosage significantly inhibited BSM contraction and voiding phenotype. Interestingly, the total urine volume at 4-h VSA did not show a difference among groups. This is because mice are water-restricted during VSA, which limits the total urine output in the 4-h VSA experiment. Thus, these data demonstrated complex competing phenotypes from C21, kidney-sourced diuresis, and bladder contraction inhibition depending on the dose.

## Discussion

We demonstrated that C21 causes off-target effects on renal function in wild-type mice not expressing any DREADD transgene, resulting in both diuresis and altered bladder storage/voiding function. The effect occurred at 1.0–3.0-mg/kg dosages as we tested but was neither apparent at 0.3 mg/kg nor in control experiments where vehicle D5W was administered. This dose-dependent effect is obvious in both awake and anesthetized studies. These off-target effects of C21 indicate that its use must be tempered with caution, and appropriate controls must be used in the renal/urological system.

DREADD-based compounds C21 and CNO were developed to function as agonists to activate engineered receptors but are “inert” to activate the endogenous receptors. This seems true because C21 and CNO did not induce an instant BSM contraction in our myography study, in which the muscarinic M3 receptor plays a major role in mediating BSM contractility. However, this does not imply that C21 and CNO are pharmacologically inert; instead, they could function as effective antagonists in blocking endogenous receptors and causing biological consequences. Both C21 and CNO are reported to bind to many endogenous G protein-coupled receptors (GPCRs) (Jendryka et al., 2019), although these antagonistic effects have not been well-explored. In a recent report, C21 injection at 0.5–1.0 mg/kg induced a robust and long-lasting increase in nigral dopaminergic neuron activity in rats, possibly through inhibiting 5-HT2 or H1 GPCRs expressed in these neurons (Goutaudier et al., 2020). Our and previous studies thus demonstrated that C21 has complex pharmacological effects in different tissues.

It is clear that C21 antagonizes muscarinic M3 receptors in the urinary bladder and, thus, inhibits bladder contraction and alters *in vivo* bladder function (Figures 2, 3). The mechanism of C21-mediated diuresis is much less clear. Muscarinic M3 receptors are also present in the renal system, and we expect that C21 also inhibits the kidney M3 receptors. However, it has been reported that the activation but not inhibition of M3 receptors increases vasodilation and renal plasma flow and causes diuresis (Wierema et al., 1997; Yun et al., 1993). Therefore, the interaction of C21 with the kidney M3 receptor and how it contributes to the diuresis effect observed need further clarification. Another possible target of C21 could be the vasopressin V2 receptor; it has been known that vasopressin V2 receptor antagonism augments water excretion without changes in renal hemodynamics or sodium and potassium excretion (Costello-Boerrigter et al., 2006). Jendryka et al. (2019) reported very modest binding competition by C21 to the vasopressin V1A receptor but did not test the V2 receptor (Jendryka et al., 2019).

In conclusion, C21 has multiple targets other than the engineered DREADDs in the renal system. It can function as an antagonist to cause significant renal/urological side effects at normal chemogenetic dosages.

## Data Availability

The raw data supporting the conclusion of this article will be made available by the authors, without undue reservation.
